# Space Use of Bumblebees (*Bombus* spp.) Revealed by Radio-Tracking

**DOI:** 10.1371/journal.pone.0019997

**Published:** 2011-05-16

**Authors:** Melanie Hagen, Martin Wikelski, W. Daniel Kissling

**Affiliations:** 1 Biological Collection, University of Bielefeld, Bielefeld, Germany; 2 Department of Migration and Immuno-Ecology, Max Planck Institute for Ornithology, Radolfzell, Germany; 3 Ecoinformatics and Biodiversity Group, Department of Bioscience, Aarhus University, Aarhus, Denmark; Royal Holloway University of London, United Kingdom

## Abstract

**Background:**

Accurate estimates of movement behavior and distances travelled by animals are difficult to obtain, especially for small-bodied insects where transmitter weights have prevented the use of radio-tracking.

**Methodology/Principal Findings:**

Here, we report the first successful use of micro radio telemetry to track flight distances and space use of bumblebees. Using ground surveys and Cessna overflights in a Central European rural landscape mosaic we obtained maximum flight distances of 2.5 km, 1.9 km and 1.3 km for *Bombus terrestris* (workers), *Bombus ruderatus* (worker), and *Bombus hortorum* (young queens), respectively. Bumblebee individuals used large areas (0.25–43.53 ha) within one or a few days. Habitat analyses of one *B. hortorum* queen at the landscape scale indicated that gardens within villages were used more often than expected from habitat availability. Detailed movement trajectories of this individual revealed that prominent landscape structures (e.g. trees) and flower patches were repeatedly visited. However, we also observed long (i.e. >45 min) resting periods between flights (*B. hortorum*) and differences in flower-handling between bumblebees with and without transmitters (*B. terrestris*) suggesting that the current weight of transmitters (200 mg) may still impose significant energetic costs on the insects.

**Conclusions/Significance:**

Spatio-temporal movements of bumblebees can now be tracked with telemetry methods. Our measured flight distances exceed many previous estimates of bumblebee foraging ranges and suggest that travelling long distances to food resources may be common. However, even the smallest currently available transmitters still appear to compromise flower handling performance and cause an increase in resting behavior of bees. Future reductions of transmitter mass and size could open up new avenues for quantifying landscape-scale space use of insect pollinators and could provide novel insights into the behavior and requirements of bumblebees during critical life stages, e.g. when searching for mates, nest locations or hibernation sites.

## Introduction

Quantifying animal space use is fundamental for understanding population processes and for developing conservation and agricultural management plans. For instance, movement of pollinators is crucial for pollen transport in wild and crop plant populations because many flowering plants rely on animals for pollination services [1], [2]. Bees are the most important taxon among animal pollinators and provide pollination services for more than one third of cropspecies worldwide [3]. Especially in areas with intensive agriculture, several solitary bee species are highly threatened [4], [5] and recently documented declines in bee diversity [6] illustrate the urgent need to improve our understanding of how insect pollinators move on a landscape scale. Population declines in bumblebee species, a highly important pollinator group [7], [8], [9], have been attributed to a reduced availability of suitable food resources in agricultural landscapes [10], [11], a reduction in nesting and hibernation sites [10], [12], competition from introduced species [13], and potential pathogen spillover from commercially reared colonies [14]. However, for most bee species the spatial dynamics of resource use at the landscape scale remain unknown [15]. A major problem that prevents understanding the space use of small organisms such as bees is that adequate long-distance tracking methodologies for such taxa have not been available (but see [2], [16]).

Bumblebees are largely confined to temperate, alpine and arctic zones of Europe, North America and Asia [17], [18]. All 250 species of bumblebees are relatively large, hairy and facultative endotherms. In Europe and North America, bumblebees are among the most important wild pollinators of crops [8]. Unfortunately, bumblebee species have declined in recent decades worldwide [19], mainly driven by land-use changes that cause reductions in the abundance of food plants [20]. Bumblebees are central place foragers and perform foraging trips between the central place (i.e. nest) and foraging patches. Theoretical models of energy expenditure and foraging behavior predict that flight distances can extend over several kilometers [21]. However, empirically it remains difficult to measure flight distances of bees. Previous studies have quantified bee movements by using (1) indirect measures such as foraging trip duration [22], [23], homing abilities [24]–[26], or modeling of maximum foraging ranges [21], or (2) direct measures such as mark-reobservation experiments [27]–[31], genetic microsatellite approaches [32]–[34], or harmonic radar [1], [35]. While some of these techniques (e.g. pollen mapping, mark-recapture, or genetic analyses) tend to measure minimum (rather than maximum) foraging distances, others (e.g. homing and feeder training experiments) often overestimate the routine foraging behavior of bees under natural conditions [36].

Despite being an established tracking technique for birds and mammals for decades, radio-tracking has only recently been used with insect pollinators, namely African carpenter bees [2] and Neotropical euglossine ‘orchid’ bees [16]. Harmonic radar (where individual bees carry small transponders that re-radiate radar transmissions) has been applied earlier [1], but the radar's range is limited (∼600 m) and affected by physical barriers such as hedges. Here we report the first use of radio-tracking of bumblebees with the aim to (1) test whether newly developed, miniaturized radio-tags can be used for studying movement paths of bumblebees, (2) investigate the effect of radio-tags on the behavior of the bumblebees, and (3) illustrate potential applications for quantifying movement behavior and space use of bumblebee sat the landscape scale. More specifically, we compare measured flight distances with published estimates from alternative techniques and theoretical models and describe the spatio-temporal movements and habitat use of one *Bombus hortorum* individual at our study site. Our results suggest that radio telemetry of bumblebees has the potential to provide new avenues for studying the flight behavior and movement paths of this important pollinator taxon, particularly once technological developments allow further significant reductions in both transmitter mass and size, and that these smaller and lighter transmitters are shown to have negligible effects on bee behavior.

## Materials and Methods

### Study time and area

The study was conducted between June-29 and July-5 2009 at the ‘Bee Marie’ conservation meadow in the vicinity of the Max Planck Institute for Ornithology at Möggingen near Radolfzell, Lake Constance region, Germany (8°59′52E and 47°45′55N latitude). The study area is a rural landscape mosaic composed of villages, meadows, fields, hedgerows and forest patches. Bumblebees were caught and transmitters were attached at the study site (‘Bee Marie’ conservation meadow).

### Study organisms

The study was conducted using workers of *Bombus terrestris* (subsp. *terrestris*) from a commercially purchased nest (NATUPOL, Sautter& Stepper GmbH) located at the study site, one worker of *B. ruderatus* and (presumably young) queens of *B. hortorum*, both caught from the wild. Whether these young queens were gynes (unmated queens) or pre-hibernation mated queens (searching for a hibernation site) remains unknown.While *B. terrestris* and *B. hortorum*are very common species in Europe that are easily found in gardens, orchards or parks, *B. ruderatus* is rather rare and distributional data are limited due to its resemblance to *B. hortorum* [37]. We assessed body size of *B. terrestris* and *B. hortorum* individuals by measuring the distance between the wing bases (intertegular (IT) span, [38]) on a sample of 5–6 individuals each. These individuals were either those bumblebees we fitted transmitters to or bumblebees of comparable size. Due to lack of specimens we measured the IT span for only one individual of *B. ruderatus*. To measure IT we used a dissecting microscope and calibrated ocular micrometers in the lab. IT span is empirically related to dry body mass, IT span  = 0.77(dry weight)^0.405^ (*R* ^2^ = 0.96; mass in mg and IT in mm, [38]), so that body mass can be estimated from IT span data. We approximated the live weight of the bumblebee individuals by using the calculated dry weight and the live:dry weight ratios as reported in the literature [39].

### Transmitter attachment

**Figure 1:**
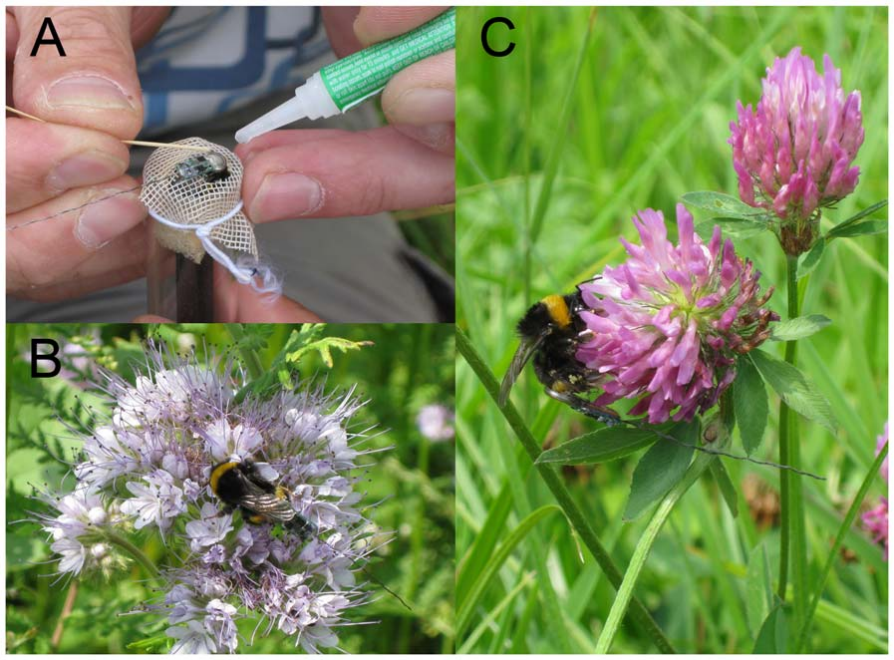
Miniaturized radio transmitters attached to bumblebees. (*a*) Transmitter attachment on a *Bombus terrestris* individual kept in a glass tube with opened gauze where the transmitter is fixed with superglue. (*b*) Nectar collecting individual of *Bombus terrestris* on *Phacelia* flower having a transmitter attached. (*c*) *Bombus terrestris* individual with attached transmitter, foraging on red clover (*Trifoliumpratense*).

We fitted transmitters to three (presumably young) *Bombus hortorum* queens, to one *B. ruderatus* worker and to four *B. terrestris* workers. For transmitter attachment, bumblebee individuals were put into a glass tube where one end was closed with gauze and the other end closed with foam (Figure 1*a*). The gauze was then partly opened with scissors so that dorsal parts of the bumblebee body were accessible but the animal was still fixed in the glass tube (Figure 1*a*). Collected individuals were fitted with small (200 mg) radio transmitters (Advanced Telemetry Systems, Isanti, MN, Series A2405, antenna length shortened to ca. 3 cm) on the dorsal upper abdomen using minute amounts of a combination of eyelash adhesive (DUO Lash Adhesive, American International Industries, Commerce, CA) and superglue (Instant Krazy Glue, Elmer's Products, Inc., OH). To keep transmitter weight to a minimum required the use of a small battery, limiting the transmitter life to a period of about seven days. We initially tested attaching the transmitter to the dorsal thorax of bumblebees, a method we previously found worked successfully with orchid bees [16]. However, bumblebees with transmitters attached in this way showed unbalanced flight behavior and we consequently abandoned this attachment method.

### Effects of transmitter attachment

To evaluate the effect of transmitter attachment we report behavioral observations on the bumblebee species. This includes observations on (1) the behavioral responses of bumblebee individuals of all species directly after transmitter attachment, (2) the movement behavior of tagged *B. terrestris* individuals at the purchased nest, and (3) detailed behavior obtained from tracking one *B. hortorum* individual queen at the landscape scale (see below ‘Spatio-temporal habitat use’).

Additionally, we quantified the effect of transmitter attachment on bumblebee flower visitation behavior. We observed individuals of *B. terrestris* (with and without transmitters) in a 3×25 m patch of sown phacelia plants (*Phaceliatanacetifolia*, Boraginaceae) at the study site and recorded visitation rate (i.e. number of *Phacelia* flower heads visited per minute) and time on flowers (foraging time [in seconds] spent on individual *Phacelia* flower heads) within time intervals of 0.5–3 minutes per individual (8 time intervals of *B. terrestris* with transmitter, 12 intervals/individuals without, both time intervals adding up to a total of 21.5 minutes observer time). We statistically compared both mean visitation rates and mean time on flowers between groups (i.e. bumblebees with transmitters vs. without) using one-tailed t-tests on log-transformed data. The two other species (*B. hortorum* and *B. ruderatus*) were not sufficiently abundant to conduct similar studies.

### Tracking

After transmitter attachment, the bumblebees were released and tracked by two people using conventional radio telemetry techniques [40]. Additionally, we located bumblebees using three aerial surveys when bumblebees were outside the detection range of people tracking on the ground. These surveys were conducted with a Cessna aircraft equipped with external receiver antennas. Whenever a signal from a transmitter was received, the location was noted with a Global Position System (GPS). Because of visual contact, the exact position, the behavior (e.g. foraging vs. moving) and the flight path of bumblebee individuals could be noted in most cases when bees were tracked on the ground. In cases of longer flight movements (e.g. >100 m), the bumblebee was followed by foot, visual landmarks were noted by the tracking person, and GPS points of the flight path were taken later. Tracking of bumblebees took place between 8 am and 8 pm.

### Flight distances and home range sizes

For each individual bumblebee we estimated the maximum flight distance (i.e. the distance between the study site and the most distant location). We compared this observed maximum flight distance with the homing distance as predicted by the Greenleaf et al. (2007) regression model [36]. This model is the only empirically derived model that predicts flight distances across bee species and is based on a log-log linear regression model between body size (i.e. intertegular span, [38]) and foraging distance according to the function log y = log a+b * log x (where y =  foraging distance, a =  constant, b =  power parameter, x =  body size of the bee). Following this model, we performed logistic regressions on the data from both bumblebee species (*B. terrestris*, *B. hortorum*) to generate the predicted distance for return of 10% of individuals (i.e. the distance Greenleaf et al. [36] defined as the "maximum homing distance". N.B. despite the wording error in Greenleaf et al. [36], maximum homing distance is correctly defined as the "loss of 90% of individuals" (Neal Williamspers. comm.)) and 50% of individuals (i.e. the distance Greenleaf et al. [36] defined as the "typical homing distance"). 'To apply these calculations, we measured body size using intertegular span (IT span) for 6 *B. terrestris* and 5 *B. hortorum* individuals (see above 'Study organisms').

Besides maximum flight distances we further estimated the home/foraging range size for all individuals with ≥5 point locations (see above ‘Tracking’). We used the minimum convex polygon [MCP] method [41] and estimated MCP home range sizes in ArcView GIS (see below). The MCP is the most common method of estimating home range sizes [41] and is constructed by connecting the outer locations to form a convex polygon, and then calculating the area of this polygon (see [42] for the specific formula). We call the bee's individual movement ranges ‘home range’ for convenience although the (presumably young) queens of *B. hortorum* could be already in a stage where they leave their natal colony and search for hibernation sites.

### Spatio-temporal habitat use

To illustrate an example of how bumblebee telemetry data can be used to study spatio-temporal habitat use, we took images from the study area from Google Earth (version 4.3.7284.3916, build date July 8, 2008, available at www.earth.google.com) and geo-referenced them using the open source software MapWindow GIS (version 4.7.4, 10/08/2009, available at www.mapwindow.org/) and the plugin Shape2Earth (version 1.46, www.shape2earth.com). These geo-referenced images (in geographic projection) were then transferred to the Geographic Information System (GIS) ArcView 3.2 (ESRI, Redlands, CA, USA) for creating a spatial landcover file in GIS format. Four major landcover types were distinguished and digitized from the Google Earth images: (1) trees (including individual trees, hedges and forest patches outside villages), (2) agricultural fields, (3) meadows, and (4) villages (including buildings, gardens and roads). Most of the digitized landcover patches were checked in our field work. For subsequent GIS analyses, we projected the landcover file with a Transverse Mercator projection and measured distances (in m) and areas of home ranges (in ha). To analyze bumblebee habitat use we chose the best tracked bumblebee individual (young *B. hortorum* queen, bee 1, Table 1, observed for >12 hours) and recorded the time it spent at each location and its behavior (resting (when the bumblebee was not moving), foraging (when the bumblebee was observed visiting flowers), or moving (when the bee flew straight from one point to the other) to quantify its spatio-temporal space use. We took all its GPS locations around the study site (*n* = 40, excluding the most distant point which was obtained by aircraft and with 1,316 m away from the study site a clear outlier in the spatial coverage and frequency distribution of distances for this individual) and intersected the GPS locations with the digitized GIS landcover types. We then calculated the proportional use of landcover types based on the observed GPS locations (note that including the outlier would only negligibly change the observed proportional habitat use of this individual) and compared it to a simulated random habitat use. To simulate random habitat use, we chose a radius of 320 m (which is the distance of the farthest observation point to the center) around the mean center of the 40 observation points and randomly located the same number of points (*n* = 40) within this radius. We performed this procedure one thousand times and calculated for each of the one thousand realizations the proportional availability of landcover types by intersecting the random point locations with the GIS landcover types. We used a Chi^2^-test (including the frequencies of all four landcover types) to test whether observed frequencies of habitat use of the radio-tracked bumblebee significantly deviate from the mean simulated random habitat use of landcover types. We then used t-tests to specifically test which observed proportional uses of landcover types (trees, fields, meadows, villages) deviate significantly from random habitat use. T-tests were repeated by weighting the locations with the time the bumblebee spent at them to see whether the temporal use of location points influenced the (purely) spatial habitat analysis. Note that an alternative analysis (Figure S1) using the study site as the central point of the radius (rather than the mean center of the 40 observation points) for locating the random points gave similar results to those presented here. We did not use the nest as the central point of the radius because the location of the natal nest was unknown and the young queen might not visit the natal nest anymore.

**Table 1:** Results from tracking individual bumblebees fitted with miniaturized radio transmitters.

ID	Species	Days^a^(count)	Locations^b^(count)	Home range size^c^ (ha)	Distance^d^(m)	Typical homing distance^e^ (m)	Maximum homing distance^e^ (m)
Bee 1	*B. hortorum*	4	41	37.69	1,316	6,682	15,824
Bee 2	*B. terrestris*	4	4	-	2,535	3,975	9,229
Bee 3	*B. ruderatus*	2	5	43.53	1,943	6,150	14,518
Bee 4	*B. hortorum*	1	9	0.25	84	6,682	15,824
Bee 5	*B. terrestris*	1	2	-	17	3,975	9,229
Bee 6	*B. terrestris*	1	3	-	351	3,975	9,229
Bee 7	*B. hortorum*	1	11	1.37	397	6,682	15,824
Bee 8	*B. terrestris*	1	2	-	1,286	3,975	9,229

a Number of days observed (from day of transmitter attachment to day of last observation).

b Number of point locations recorded from radio tracking (including site of transmitter attachment).

c Home range size (for individuals with ≥5 points) using the minimum convex polygon (MCP) method.

d Distance between site of transmitter attachment and most distant location revealed from radio tracking.

e calculated with model from Greenleaf et al. 2007.

### Data Analysis

All statistical analyses were done with R (version 2.9.0, R Development Core Team 2009). For the GIS analyses in ArcView, we used the extension ‘Nearest features’ (version 3.8b) to measure the distances between the site of transmitter attachment (i.e. study site) and the most distant location, the extension ‘Convex hulls around points’ (version 1.24) to estimate MCP home range sizes, the extension ‘Weighted Mean of Points’(version 1.2c) to calculate the mean center of the 40 observation points,and the extension ‘Random Point Generator’ (version 1.3) to generate the sets of random points for the habitat analysis. All extensions are freely available from Jenness Enterprises [43].

## Results

### Study organisms

The mean (±SD) intertegular span of *B. terrestris* workers was measured to be 4.92±0.25 mm (*n* = 6), and following the calculations by Cane [38] the mean dry weight can be estimated to be ca. 97.5 mg. The mean (±SD) intertegular span of of*B. hortorum* queens was 5.80±0.49 mm (*n* = 5) with an estimated mean dry weight of about 146 mg. The intertegular span of the single *B. ruderatus* specimen was 5.63 mm with an estimated dry weight of about 136 mg. The scarce data on live:dry mass ratios suggest that the live masses of bumblebees (based on data for males and queens of *B. terrestris*) are about two- to threefold higher than their dry weights[39], even though live mass is highly variable due to e.g. nectar intake. Given these values, the life weight of *B. terrestris*workers in this study can be estimated to be 200–300 mg,the life weight of *B. hortorum*queens around 300–450 mg, and the life weight of *B. ruderatus* workers around 270–400 mg. Hence, the transmitter weight is ca. 66–100 % of the life weight of a *B. terrestris* worker, ca. 44–66% of the life weight of a *B. hortorum* queenand about 50–74% of the life weight of a *B. ruderatus* worker.

### Effects of transmitter attachment

We successfully fitted miniaturized radio transmitters to eight bumblebee individuals of which four were *B. terrestris* workers (from the purchased nest), three *B. hortorum* queens (from the wild) and one a *B. ruderatus* worker (from the wild). The tagged bumblebees usually took off within 1 minute after fitting the radio transmittersand then flew to a nearby bush or tree where they spent the next ½ to 2 hours cleaning themselves (probably as a consequence of handling and attaching the transmitter). After the cleaning period, bumblebees flew larger distances (>100 m) to suitable foraging patches. Bumblebee individuals of *B. terrestris* were able to enter the purchased nest with the transmitters attached and we could see them moving around within the nest cells.The radio-tracking data of one *B. hortorum* individual further indicated that resting periods during flight movements can be long (>45 min, see below), suggesting that the transmitter weight may incur significant energetic costs.

**Figure 2:**
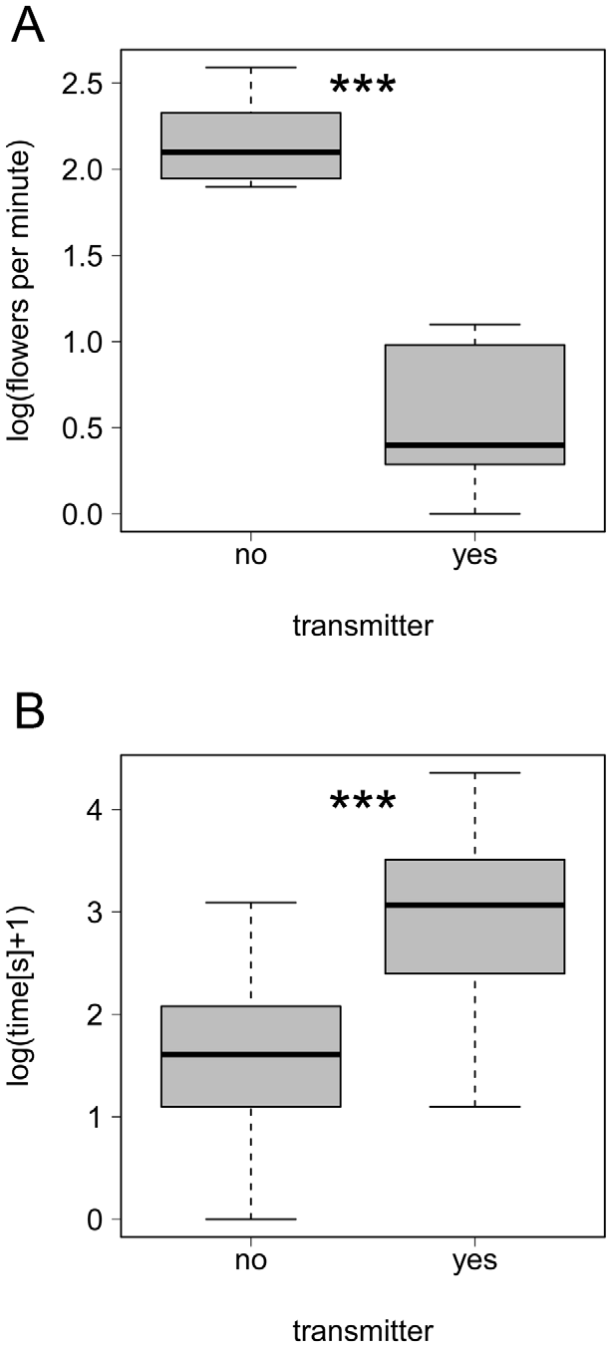
Effect of transmitter attachment (yes/no) on foraging behavior of *Bombus terrestris*. (*a*) Visitation rates (number of *Phacelia* flower heads visited per minute), and (*b*) time on flowers (foraging time spent on individual *Phacelia* flower heads). *** indicates significant differences between groups at *p*<0.001 (see text).

All individuals were able to feed successfully on flowers when transmitters were attached (Figure 1). However, transmitter attachment showed an effect on foraging behavior of *B. terrestris* (Figure 2). Bumblebees with transmitters had significantly lower flower visitation rates (Figure 2*a*; one-tailed t-test: t = 11.7, df = 213, *p*<0.001) and spent significantly more time foraging on individual flower heads (Figure 2*b*; one-tailed t-test: t = 11.2, df = 18, *p*<0.001) than individuals without transmitters. We were unable to quantify whether this also means that foraging efficiency (i.e. nectar consumption per time) is reduced, or whether it simply reflects a difference in flower handling. Similar observations on the effect of transmitter attachment could not be carried out for *B. hortorum*or *B. ruderatus* because they were not abundant enough at the study site.

### Flight distances and home range sizes

**Figure 3:**
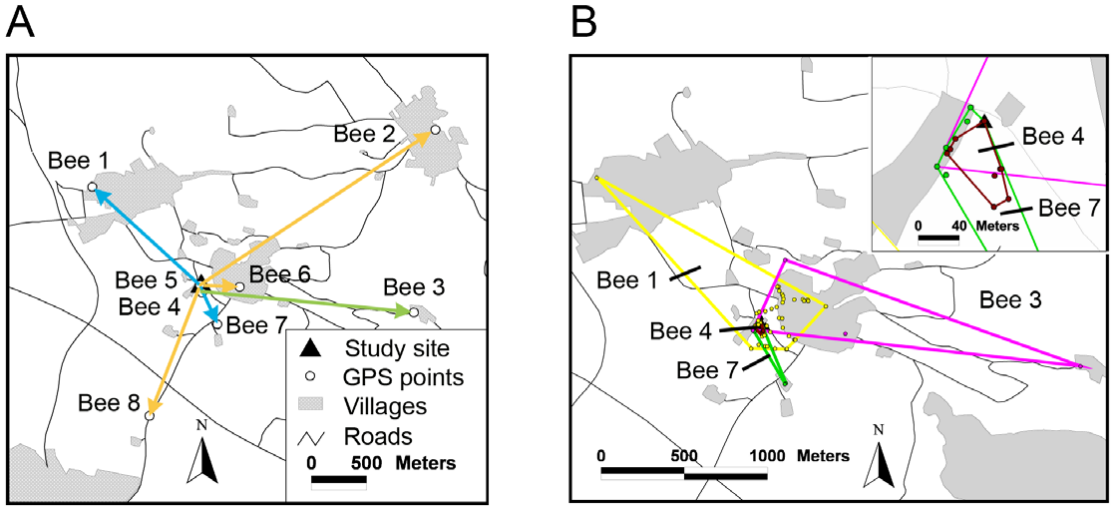
Flight distances and home ranges of bumblebees. (*a*) Maximum flight distances (bees 1–8) recorded from radio-tracking. Blue arrows: *Bombus hortorum* individuals, green arrow: *B. ruderatus*, orange arrows: *B. terrestris*, (for distance estimates see Table 1). Note that bee 4 (*B. hortorum*) and bee 5 ( *B. terrestris*) are not illustrated with arrows due to their short flight distances. (*b*) Estimated home ranges (minimum convex polygons[MCP] and observation points) for individuals with ≥5 point locations(bee 1, 4, 7 *B. hortorum*, bee 3 *B. ruderatus*; see Table 1 for estimated home range sizes). Different colors represent different individuals (bee 1, 3, 4 and 7). The inset shows a more detailed view of the study site where the observation points of bee 4 (brown) and bee 7 (green) are plotted. Note that four observation points of bee 4 (lying on the MCP line) are covered by observation points of bee 7.

The eight bumblebee individuals were observed between 1–4 days yielding a total of 77 point locations (2–41 per individual, Table 1). Seven locations (including all distances >1 km) were obtained during the three Cessna airplane overflights. The maximum flight distances (Figure 3*a*) were on average 991±927 m (mean ± SD, median  = 842 m). The largest distances that we recorded were2,535 m, 1,316 m and 1,943 m for*B. terrestris, B. hortorum and B. ruderatus*, respectively (Table 1). In comparison, the typical homing distances predicted by the regression model of Greenleaf et al. [36] using the measured IT data were 3,975 m for *B. terrestris*, 6,793 m for *B. hortorum* and 6,150 m for *B. ruderatus*, clearly larger than our empirically measured flight distances (Table 1). The maximum homing distances predicted by this model were even larger (9,228 m for *B. terrestris*, 15,824 m for *B. hortorum* and 14,518 m for *B. ruderatus* , Table 1).

Home ranges were estimated and mapped for all bumblebee individuals with >5 GPS locations (Figure 3*b*). This was possible for 4 individuals, three of which were (possibly young) queens of*B. hortorum* and one a *B. ruderatus* worker. The estimated MCP home range sizes varied from 0.25–43.53 ha (Table 1) and indicated that bumblebee individuals can use large areas for foraging, even within a few days(Figure 3*b*).

### Spatio-temporal habitat use

**Figure 4:**
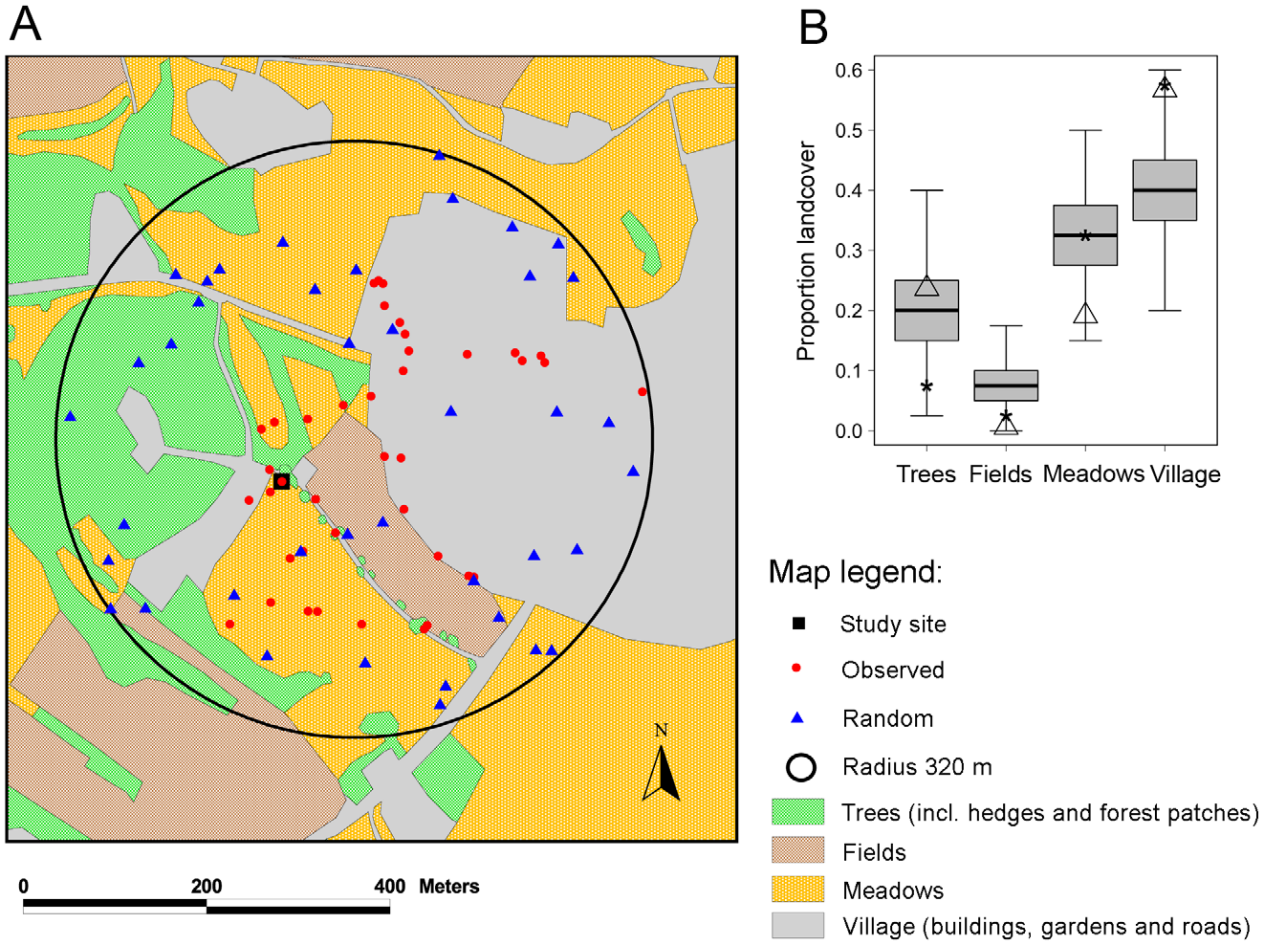
Habitat use of one bumblebee individual (*Bombus hortorum*, bee 1, **Table 1** **).** (*a*) Map of major landcover types with observed bumblebee locations (red points, *n* = 40) and one realization of random points (blue triangles, *n* = 40) within a radius of 320 m around the mean center of the 40 observation points. A total of one thousand realizations of random points (each with*n*  = 40) were used to simulate random habitat use around the mean center of the 40 observation points. (*b*) Proportional use of four landcover types in comparison to simulated random habitat use (1000 simulations). Boxplots indicate simulated random habitat useas measured by intersecting landcover types withone thousandrealizations of 40 random points. Asterisks denote the proportional use of landcover types as observed from radio-tracked bumblebee locations (red points in (*a*)). Triangles show the proportional use of landcover types when locations are weighted by the time the bee spent at them. Statistical results of testing differences between observed and simulated random habitat use are provided in Table 2.

The illustrative example of a quantitative habitat analysis of one *B. hortorum* individual (bee 1, Table 1) showed that the proportional habitat use of this bumblebee deviated significantly from the mean simulated random habitat use of landcover types in the study area (Chi^2^ = 11.55, df = 3, *p*<0.01, Figure 4). In particular, agricultural fields were used less often and villages more often than expected by random simulation (Figure 4*b*). Meadows were only used more often than expected by random simulation when time was accounted for and trees (including hedges and forest patches) were used less often except when time was accounted for (Figure 4*b*).The observed proportional uses of landcover types deviated significantly from random habitat use in all cases except for the observed use of meadows based on spatial locations only (Table 2).

**Table 2:** Proportional use of landcovertypes of one bumblebee individual (*Bombus hortorum*, bee 1, Table 1).

	Proportional use of landcover types		T-test		
Landcover types	Observed	Simulated (mean ± SD)	*t*	df	*p*
*Spatial locations only*					
Trees	0.075	0.201 (±0.064)	62.54	999	<0.001
Fields	0.025	0.076 (±0.042)	38.33	999	<0.001
Meadows	0.325	0.324 (±0.073)	−0.62	999	0.535
Villages	0.575	0.399 (±0.078)	−71.12	999	<0.001
*Locations weighted by time*					
Trees	0.237	0.201 (±0.064)	−17.45	999	<0.001
Fields	0.006	0.076 (±0.042)	52.88	999	<0.001
Meadows	0.191	0.324 (±0.073)	57.79	999	<0.001
Villages	0.567	0.399 (±0.078)	−67.87	999	<0.001

Comparisons are made between observed bumblebee locations (spatial locations only, locations weighted by the time the bee spent at them) versus simulated random habitat use. Simulated random habitat proportions (mean±SD) were obtained from one thousand realizations of random points (each with*n*  = 40) (compare Figure 4).

**Figure 5:**
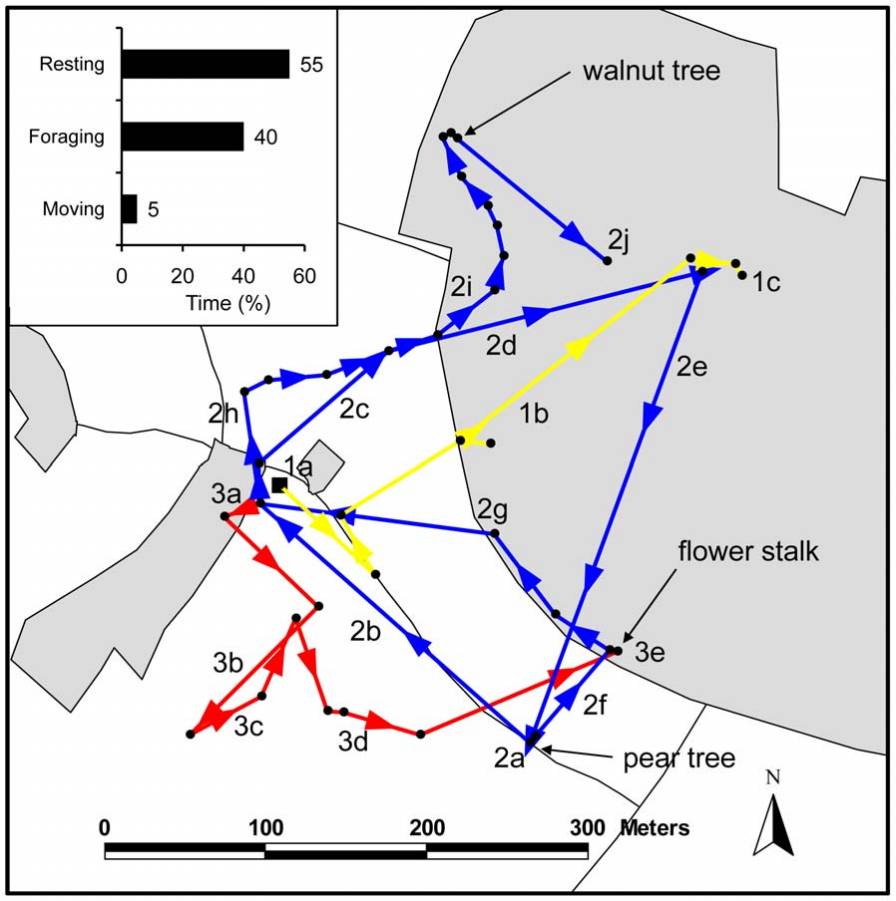
Detailed movement trajectories of one *Bombus hortorum* individual (bee 1, see **Table 1**) followed over a time period of >12 hours (within 2 days) with 3 continuous tracking periods. Trajectory 1 (yellow line, order from 1a to 1c) is the first tracking period from 1–3:45 pm (165 min), starting from the study site (black square) after transmitter attachment on the first day (June-30). Trajectory 2 (blue line, from 2a to 2j) is the second tracking period from 8:45 am to 2:55 pm (370 min) on the following day (July-1), and trajectory 3 (red line, from 3a to 3e) is the third tracking period in the afternoon of the same day from 4:25–7:50 pm (205 min). The inset shows the percentages of total time spend for three different behavioral categories (resting, foraging, and moving). Note that the bumblebee rested for long time periods (>45 min) on a pear tree (105+80 min), a walnut tree (95 min), and a flower stalk (60 min + subsequent overnight stay). Flight times between points were rather short (usually <1 min).

We were able to directly follow this *B. hortorum* individual (bee 1, Table 1) by foot for >12 hours within 2 days with 3 continuous tracking periods of 165, 370 and 205 minutes, respectively. The bumblebee spent approximately half of the recorded time (48%) inside and the other half (52%) outside the village boundaries (Figure 5). Movements between the village and the surrounding landscape were frequent and the bumblebee returned several times to the same localities, either to feed on flower resources or to rest (e.g. on trees, in the meadow, or on a flower stalk). Interestingly, >50% of the recorded time was spent resting (Figure 5 inset) whereas 40% of the time was used for foraging. Only 5% of the recorded time was spent for flight movements. Two trees and one flower stalk were used for resting periods of at least 45 min length (Figure 5) and the bumblebee stayed on the flower stalk overnight (this overnight stay was not included in our time estimates).

## Discussion

Our results represent the first successful use of radio-tracking methodology for studying movement behavior and space use of bumblebees. Individuals of *B. terrestris*, *B. hortorum* and *B. ruderatus* flew distances up to 2.5 km and used large areas (0.25–43.53 ha) in a relatively short time period (1–4 days). The spatio-temporal habitat use at a landscape scale and the detailed movement trajectories of one *B. hortorum* queen further showed that this bumblebee preferentially selected certain habitat types and repeatedly visited prominent landscape structures (e.g. trees) and flower patches for resting and foraging, respectively. Such constancy in route following behavior confirms earlier suggestions that experienced individuals can return to the same site each day for weeks, visiting the same clumps of plants in very similar sequences (e.g. [1], [44]–[47]). However, the low sample size of our study means we should be cautious in generalizing our findings, and future studies are needed to substantiate our results.

### Effects of transmitter attachment

Although we successfully used radio-tracking methodology to study patterns of movement and space use in bumblebees, we found that transmitter attachment caused detectable effects on bumblebee behavior. Bumblebees rested and cleaned for ½ to 2 hours after transmitter attachment, a behavior that is rather unusual for bumblebees and probably related to the handling and attachment of the transmitters. Furthermore, the one *B. hortorum* individual which we observed for >12 hours showed long (>45 min) resting periods (e.g. on trees), a result that could be taken to indicate that metabolic costs are increased when transmitters are attached. However, a study of male *B. terrestris* in the lab in completely constant and predictable climates of test chambers with unlimited food supply found that only 40% of the daytime was used for flight movements, and consequently 60% for resting [48]. To what extend bumblebee queens, males and workers naturally rest during their daily flight movements in the wild has never been quantified, so we have no benchmark against which our results can be evaluated.

Our results further demonstrate that the foraging behavior of bumblebees is influenced by transmitter attachment. Radio-tagged *B. terrestris* individuals exhibited significantly lower rates of flower visitation and spent significantly longer visiting each flower than bees without transmitters. Whilst we did not measure the foraging efficiency (nectar consumption per unit time) of tagged bees, it seems likely that carrying a transmitter of 66–100% of the bee's own body mass affects flight performance and hence energy usage. Further studies could empirically test whether nectar consumption of bumblebees is affected by carrying transmitters, for example using calibrated feeder experiments in a laboratory flight room [49]. We note that our foraging assessment was only possible for *B. terrestris* because *B. hortorum* and *B. ruderatus* were not abundant enough at the study site. As transmitter mass is constant (200 mg), it seems likely that transmitter attachment would have a less significant effect on the foraging behaviour of bees larger than *B. terrestris* workers (i.e. >200–300 mg live weight).

The transmitter weight was estimated to be about 44–100 % of the body weight of the bumblebees used in this study. Thus, especially for the smaller workers of *B. terrestris* it might have consequences for their metabolism (i.e. increased energetic costs). Although bumblebees are known to be able to carry such heavy loads (e.g.nectar and pollen loads up to 100 % of their body weight [44]), the attachment of the radio transmitters could additionally have additive, long term effects. Carrying a transmitter produces energetic costs and thus reduces the amount of pollen and nectar that can be harvested during a foraging bout. Hence, our results on e.g. maximum flight distances of bumblebees should be considered as conservative estimates and further studies on the energetic consequences of carrying transmitters are clearly needed, especially for small-bodied ectothermic insects.

### Flight distances and home range sizes

Although theoretical models [21] predict bumblebee foraging distances up to several kilometers away from the nest, empirical information is scarce and most measured foraging distances are below 1 km [1], [36]. These foraging distances are typically only measured for workers, and studies on flight behavior and space use of males and queens are largely absent (see below). Generally, depending on different methods, the observed foraging distances of workers vary greatly, with rather short observed foraging distances between 300 to 800 m for some marking-reobservation experiments[27], [28] (exceptions: [15], [30], [31]), harmonic radar [1], and genetic analysis studies[32], [33], however other approaches (like homing experiments [25] and mathematical modeling [21]) suggest foraging distances of up to 10 km. Mark-reobservation experiments [15], [30], [31] like new mass-marking experiments [15] with *B. terrestris* have recently shown that foraging distances of bumblebees can easily extend beyond 1 km. Kreyer and colleagues [30] found *B. terrestris* workers foraging up to 2.2 km far from their nests, using a mark-recapture method. Our results from radio-tracking support these findings and suggest that workers might commonly travel long distances to food resources even when food is locally abundant.

While most studies on flight distances are carried out on workers, we found one study investigating dispersal distances of queens using a genetic micro satellite approach[50]. Lepais et al. [50] found (*B. pascuorum* and *B. lapidarius*) queens to travel distances of 3–5 km emphasizing the importance of queen dispersal for gene flow in bumblebee species. Using a similar approach, Kraus et al. [51] found male *B. terrestris* to fly distances of 2.6–9.9 km suggesting that male dispersal also plays a vital role in maintaining gene flow between populations. We found queens of *B. hortorum* to fly up to 1.3 km within a few days but whether these flights are related to dispersal or foraging remains unclear. The shorter flight distance that we report (compared to [50]) would not be surprising if these estimates are foraging rather than dispersal distances. The dispersal distances reported by Lepais et al. [50] include several dispersal steps between departure at the old nest and establishment of a new nest, i.e. the searches for mates, hibernating places and nest sites, and the foraging range.

Measures of flight distances can thus quantify very different behaviors of bees. While we measured foraging flights of workers of *B. terrestris* and *B. ruderatus* and maybe a mixture of foraging and dispersal flights of *B. hortorum* queens, the flight distances predicted by the model of Greenleaf et al. [36] (Table 1) refer to the homing behavior of bees. Homing experiments measure the ability of the bee to find home rather than daily foraging distances and therefore are not necessarily representative of routine distances travelled by individual bees [36]. However, in the absence of other empirically derived models for predicting flight distances of bee species, the formula from Greenleaf et al. [36] represents the only benchmark against which measured flight distance can be evaluated across bee species. We suggest that a promising area for future research could be to develop predictive models of flight distances separately for castes (workers, males, queens) and behavioral types (daily foraging, homing, dispersal).

Non-parasitic bumblebees (like other non-parasitic bee species) provision their broods by central place foraging, which means they gather pollen and nectar from flowers in the surrounding area and bring it back to a central nest. The foraging and home range of bees is thus a fundamental aspect of their ecology, as it determines the area of the habitat that an individual or a colony can exploit. However, home range sizes of bees have rarely been quantified. For male euglossine bees, which are known to fly very long distances (up to 24 km, [16], [24]), Wikelski et al. [16] estimated MCP home range sizes of 4–700 ha (mean±SD: 45±40 ha) in a rainforest environment in Panama. The bumblebee species studied here have smaller home range sizes than the euglossine bees. This probably reflects the extraordinary ability of male euglossine bees to fly large distances to find mating partners, orchids and other food plants which are spatially rare in tropical forests [16]. We are not aware of any other study that has quantified MCP home range sizes of bees, but depending on caste (worker, queen, male) and species (solitary, social), home range size should vary widely. Bumblebee home range size could be considerably larger than our estimates both because current radio transmitters only allow for short observation times due to power constraints, and also because the relatively large size and mass of current transmitters may limit their flight potential.

### Spatio-temporal habitat use

On a landscape scale, the radio-tracked *B. hortorum* individual spent a large proportion of its time in the village, and this was significantly more time than expected from habitat availability around the study site. During the time of study, gardens within villages provided a large diversity and abundance of flower resources while agricultural fields were largely depleted. At other times of the year, flower resource availability might change and bumblebee individuals are likely to track the spatio-temporal dynamics of resource availability [52]–[54]. Thus, bees could spend more time in agricultural fields during other times of the year when mass flowering of plants dominates the forage landscape [15], [55]. However, urban areas have the potential to be important pollinator reservoirs, especially if flower species diversity and habitat heterogeneity are maintained and enhanced through sustainable urban planning [56]. Several studies in Europe and America suggest that urbanized areas in general provide appropriate and abundant resources that bees need for survival and reproduction [57]–[60]. Many ornamental garden flowers bloom for relatively long periods and urban gardens might thus provide a relatively constant source of pollen and nectar [60].

During our observations we did not see young queens to visit the natal nest or actively search for a hibernation site. Thus, these individuals might have been in an intermediate stage between helping their natal colony with food provisioning [44] and searching for potential mates and hibernation sites. If so, the location of the natal nest could thus have confined the flight range of the young queen to garden areas. Overall, knowledge about space use and flight ranges of queens is scarce and the radio-tracking methodology could greatly help to improve our basic knowledge of queen behavior.

### Outlook

We found bumblebee individuals of *B. terrestris, B. hortorum* and *B. ruderatus* to fly successfully with transmitters of 200 mg which suggests that space use of large-bodied bumblebees (e.g. large workers or queens) can be studied with radio-tracking methodology. However, compared to the body size of bumblebees (workers 40–600 mg, queens up to 850 mg) current transmitters are still sufficiently large and heavy (200 mg) that they have been shown to affect foraging behavior, are likely to affect flight performance and/ or energetics and so might have fitness consequences for bumblebees. Future technological advances are likely to reduce transmitter weights further and hence will open up exciting avenues for studying flight behavior and movement paths of rather small-sized insect pollinators. This could have important implications for conservation and agriculture, especially for assessing ecosystem consequences of pollinator declines. We also see great potential for better understanding the basic biology of bees, e.g. the spatial behavior and requirements of queens, when searching for males, nest locations or hibernation sites.

## Supporting Information

Figure S1 **Habitat use of one bumblebee individual (** ***Bombus hortorum*** **, bee 1,** **Table 1** **).** (*a*) Map of major landcover types with observed bumblebee locations (red points, *n* = 40) and one realization of random points (blue triangles, *n* = 40) within a radius of 410 m around the study site. A total of one thousand realizations of random points (each with*n*  = 40) were used to simulate random habitat use around the study site. (*b*) Proportional use of four landcover types in comparison to simulated random habitat use. Box plots indicate simulated random habitat useas measured by intersecting landcover types with the one thousand realizations of 40 random points. Asterisks denote the proportional use of landcover types as observed from radio-tracked bumblebee locations (red points in (*a*)). Triangles show the proportional use of landcover types when locations are weighted by the time the bee spent at them.
(DOC)
