# Supplementary material for: Space Use of Bumblebees (Bombus spp.) Revealed by Radio-Tracking
Source: PLoS One. 2011 May 16;6(5):e19997. doi: 10.1371/journal.pone.0019997 (PMC3095635; doi:10.1371/journal.pone.0019997)

**Figure S1. Habitat use of one bumblebee individual (*Bombus hortorum*, bee 1, Table 1).** (*a*) Map of major landcover types with observed bumblebee locations (red points, *n* = 40) and one realization of random points (blue triangles, *n* = 40) within a radius of 410 m around the study site. A total of one thousand realizations of random points (each with *n* = 40) were used to simulate random habitat use around the study site. (*b*) Proportional use of four landcover types in comparison to simulated random habitat use. Boxplots indicate simulated random habitat use as measured by intersecting landcover types with the one thousand realizations of 40 random points. Asterisks denote the proportional use of landcover types as observed from radio-tracked bumblebee locations (red points in (*a*)). Triangles show the proportional use of landcover types when locations are weighted by the time the bee spent at them.


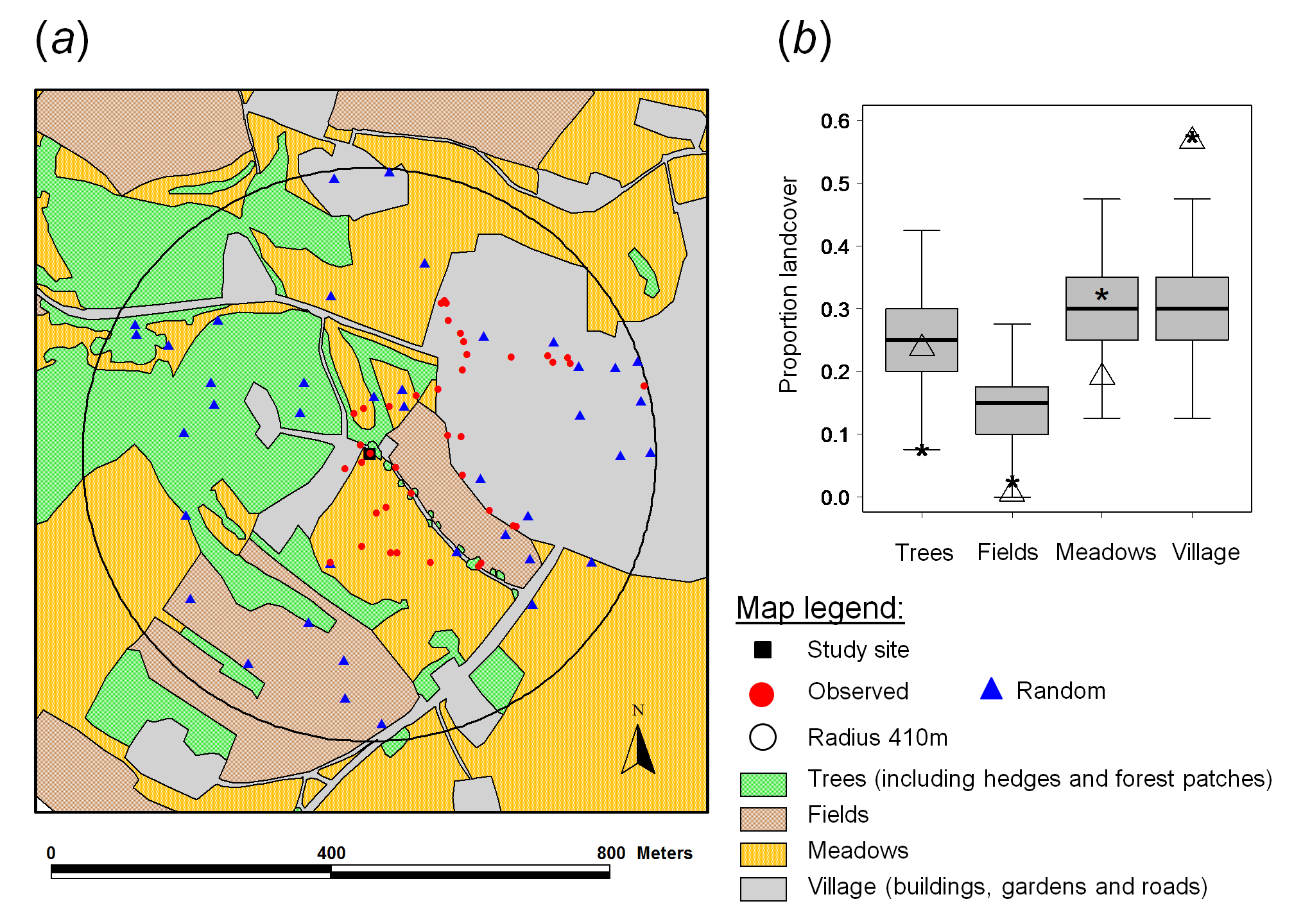

Supplement: Figure S1 — Habitat use of one bumblebee individual ( Bombus hortorum , bee 1, Table 1 ). (a) Map of major landcover types with observed bumblebee locations (red points, n = 40) and one realization of random points (blue triangles, n = 40) within a radius of 410 m around the study site. A total of one thousand realizations of random points (each withn = 40) were used to simulate random habitat use around the study site. (b) Proportional use of four landcover types in comparison to simulated random habitat use. Box plots indicate simulated random habitat useas measured by intersecting landcover types with the one thousand realizations of 40 random points. Asterisks denote the proportional use of landcover types as observed from radio-tracked bumblebee locations (red points in (a)). Triangles show the proportional use of landcover types when locations are weighted by the time the bee spent at them. (DOC) [file pone.0019997.s001.doc]
